# Clinical and psychological repercussions of videolaparoscopic tubal ligation: observational, single cohort, retrospective study

**DOI:** 10.1590/1516-3180-2014-1326687

**Published:** 2014-09-02

**Authors:** Daniel Spadoto Dias, Rogério Dias, Jorge Nahás-Neto, Eliana Aguiar Petri Nahás, Nilton José Leite, Flávia Neves Bueloni-Dias, Waldir Pereira Modotti

**Affiliations:** I PhD. Attending Physician, Department of Gynecology and Obstetrics, Faculdade de Medicina de Botucatu (FMB), Universidade Estadual de São Paulo (Unesp), Botucatu, São Paulo, Brazil; II PhD. Adjunct Professor and Chairman of the Gynecological Endoscopy and Family Planning Sector, Department of Gynecology and Obstetrics, Faculdade de Medicina de Botucatu (FMB), Universidade Estadual de São Paulo (Unesp), Botucatu, São Paulo, Brazil; III PhD. Clinical Assistant Professor, Department of Gynecology and Obstetrics, Faculdade de Medicina de Botucatu (FMB), Universidade Estadual de São Paulo (Unesp), Botucatu; and Clinical Vice-Director of the Clinics Hospital (HC) of Botucatu, Botucatu, São Paulo, Brazil; IV PhD. Adjunct Professor, Department of Gynecology and Obstetrics, Faculdade de Medicina de Botucatu (FMB), Universidade Estadual de São Paulo (Unesp), Botucatu, São Paulo, Brazil; V PhD. Collaborating Professor of Postgraduate Program on Gynecology, Obstetrics and Mastology, Faculdade de Medicina de Botucatu (FMB), Universidade Estadual de São Paulo (Unesp), Botucatu; and Clinical Director of Instituto de Atendimento à Mulher (IAM), Assis, São Paulo, Brazil

**Keywords:** Family planning services, Laparoscopy, Menstruation disturbances, Sterilization, reproductive, Sterilization, tubal, Serviços de planejamento familiar, Laparoscopia, Distúrbios menstruais, Esterilização reprodutiva, Esterilização tubária

## Abstract

**CONTEXT AND OBJECTIVE:**

Tubal ligation is one of the most commonly used contraceptive methods worldwide. Since the controversy over the potential effects of tubal sterilization still continues, this study aimed to evaluate the clinical and psychological repercussions of videolaparoscopic tubal ligation.

**DESIGN AND SETTING:**

Observational, single cohort, retrospective study, conducted in a tertiary public hospital.

**METHODS:**

A questionnaire was applied to 130 women aged 21-46 years who underwent videolaparoscopic tubal ligation by means of tubal ring insertion or bipolar electrocoagulation and sectioning, between January 1999 and December 2007. Menstrual cycle interval, intensity and duration of bleeding, premenstrual symptoms, dysmenorrhea, dyspareunia, noncyclic pelvic pain and degree of sexual satisfaction were assessed in this questionnaire. Each woman served as her own control, and comparisons were made between before and after the surgical procedure and between the two techniques used.

**RESULTS:**

The clinical and psychological repercussions were significant, with increases in bleeding (P = 0.001), premenstrual symptoms (P < 0.001), dysmenorrhea (P = 0.019) and noncyclic pelvic pain (P = 0.001); and reductions in the number of sexual intercourse occurrences per week (P = 0.001) and in libido (P = 0.001). Women aged ≤ 35 years at the time of sterilization were more likely to develop menstrual abnormalities. The bipolar electrocoagulation method showed greater clinical and psychological repercussions.

**CONCLUSION:**

Regardless of the technique used, videolaparoscopic tubal ligation had repercussions consisting of increased menstrual flow and premenstrual symptoms, especially in women aged ≤ 35 years, and also had a negative influence on sexual activity.

## Abstract (pt)

**CONTEXTO E OBJETIVO:**

A ligadura tubária é um dos métodos contraceptivos mais utilizados em todo o mundo. Como a controvérsia sobre seus possíveis efeitos ainda continua, o objetivo deste estudo foi avaliar as repercussões clínicas e psíquicas da laqueadura videolaparoscópica.

**TIPO DE ESTUDO E LOCAL:**

Estudo observacional de coorte único, retrospectivo, conduzido em hospital público terciário.

**MÉTODOS:**

Foi aplicado um questionário a 130 mulheres, entre 21-46 anos, submetidas à ligadura tubária videolaparoscópica, pelas técnicas de eletrocoagulação bipolar/secção ou inserção do anel tubário, entre janeiro de 1999 e dezembro de 2007. Nesse questionário avaliou-se: intervalo do ciclo menstrual, intensidade e duração do sangramento, sintomas pré-menstruais, dismenorreia, dispareunia, dor pélvica não cíclica e grau de satisfação sexual. Cada mulher serviu como seu próprio controle, foi realizada análise comparativa entre os períodos pré- e pós-cirúrgico e entre as duas técnicas utilizadas.

**RESULTADOS:**

As repercussões clínicas e psicológicas mostraram-se significativas, com aumento de sangramento (P = 0,001), de sintomas pré-menstruais (P < 0,001), dismenorreia (P = 0,019), dor pélvica não cíclica (P = 0,001), e redução no número de relações sexuais por semana (P = 0,001) e na libido (P = 0,001). Mulheres com idade ≤ 35 anos, no momento da laqueadura, mostraram-se mais propensas a desenvolverem alterações menstruais. A técnica de eletrocoagulação bipolar mostrou maiores repercussões clínicas e psíquicas.

**CONCLUSÃO:**

A ligadura tubária videolaparoscópica, independentemente da técnica, repercutiu com aumento do fluxo menstrual e de sintomas pré-menstruais, principalmente em mulheres com idade ≤ 35 anos, tendo também influenciado negativamente na atividade sexual.

## INTRODUCTION

Maternal mortality is one of the great worldwide challenges in medicine. It is considered to be a major indicator in public health and is directly associated with socioeconomic factors and public healthcare provision. There are many causes of death or wors ening of preexisting diseases during pregnancy and the puerpe rium. Therefore, family planning has become highly important in preventing maternal and infant morbidity and mortality, particu larly among women at high reproductive risk.^1^

Tubal ligation is one of the most commonly used contraceptive methods all over the world. The number of women currently practicing contraception in Brazil is extremely high, reaching up to 80% of those in stable unions. According to the last Brazilian National Survey on Demography and Health (PNDS), conducted in 2006, nearly all respondents controlling their fertility were using modern contraceptive methods and 29% of the women living in a relationship were sterilized. It was noteworthy that 10.9% of the respondents who had not committed themselves to a stable union had chosen tubal ligation as a birth control method.^2^

The number of women undergoing tubal ligation has become larger and larger. As a result, the complications that might be associated with this procedure (menstrual and hormonal abnormalities, and adverse effects on physical and mental health) have been widely investigated. Williams et al., in 1951, were the first to express concern about the clinical repercussions of tubal ligation, hypothesizing that sterilization might increase a woman's risk of abnormal bleeding.^3^ Since then, the existence of a post-tubal ligation syndrome has been debated. 

There is no clear consensus on the symptoms and signs of this syndrome, which remains ill-defined. While some authors have merely described it as abnormal uterine bleeding and/or pelvic pain, others have correlated it with exacerbated premenstrual symptoms (headache, dizziness and irritability), menstrual cycle irregularity (dysfunctional endometrial bleeding, metrorrhagia, excessive bleeding, polymenorrhea, intermenstrual bleeding and amenorrhea), dysmenorrhea, pelvic pain and changes to sexual behavior and psychoemotional state.^4^^-^ 18 An association with hysterectomy has also been suggested, but its physiological mechanism remains unclear.^3^^,^ 19 ^-^ 27

Occurrences of post-tubal ligation syndrome seem to be unpredictable, and the syndrome may present in various forms depending on a series of combined factors such as pre-sterilization menstrual pattern, previously used contraceptive methods, age, operative technique used and even associated diseases.^3^^,^ 5 ^,^ 9 ^,^ 10 ^,^ 12 ^-^ 14 ^,^ 16 ^-^ 18 ^,^ 25 ^,^ 27 ^-^ 37 The incidence of complications secondary to tubal ligation varies greatly (15-40%), and the frequency of menstrual abnormalities after sterilization has been reported to range from 2.5% to 60%.^12^^,^ 38 These conflicting data can be explained by the lack of uniformity in classifying this syndrome.

Since controversy over the potential effects of tubal sterilization continues, the purpose of this study was to assess the clinical and psychological repercussions of videolaparoscopic tubal ligation. 

## OBJECTIVE

To evaluate, through a preestablished questionnaire, the clinical and psychological repercussions in women undergoing videolap aroscopic tubal ligation. 

## METHODS

**Figure 1:**
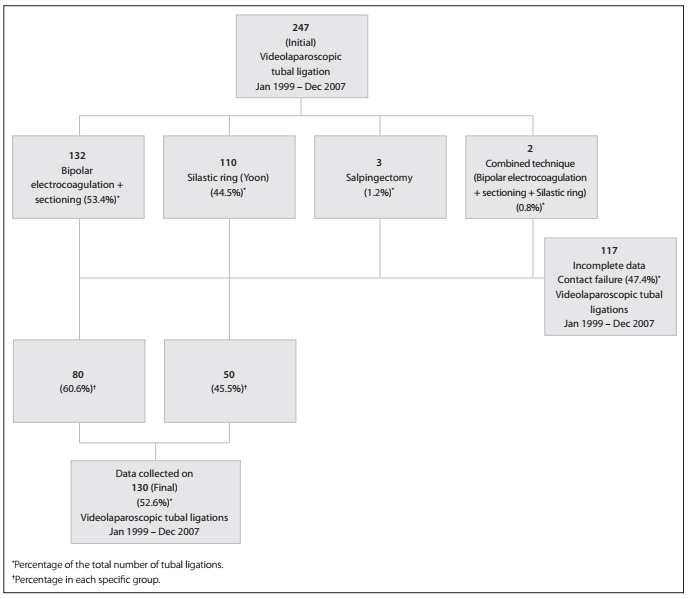
Distribution of the patients who underwent videolaparoscopic tubal ligation at the Family Planning and Gynecological Endoscopy Sector, FMB/Unesp, between January 1999 and December 2007, according to the method used.

A total of 247 women who underwent surgical laparoscopic steril ization that was followed up at the Gynecological Endoscopy and Family Planning Sector of Faculdade de Medicina de Botucatu, Universidade Estadual de SÃ£o Paulo (FMB-Unesp), between January 1999 and December 2007, were initially assessed. A sin gle cohort of 130 patients who underwent interval laparoscopic sterilization and for whom complete data were available was enrolled in this retrospective cross-sectional study (Figure 1). Approval from the institution's Research Ethics Committee and informed consent from all individuals were obtained. 

At the time of tubal ligation, all subjects except those at reproductive risk were 25 years of age or older or had at least two living children. An interval of at least 60 days was observed between manifestation of the desire for sterilization and implementation of tubal ligation, so that counseling and detailed information could be provided to the patients. Adequate information about the contraceptive methods available and the possibility of permanent sterilization of the partner (vasectomy) were also presented to all the women. Routine informed consent for voluntary permanent sterilization was obtained from each woman and this declaration was signed together with a witness, preferably the husband (Brazilian law 9263 of January 12, 1996).^39^ Patients who refused to participate and those for whom only incomplete data were available were excluded from the study. 

Potential subjects were identified through the registration system of the Clinics Hospital of FMB-Unesp and were invited to participate in the study. Visits to the hospital were then scheduled for administration of the study questionnaire (**Appendix 1**). Women who missed the visit or could not be successfully reached were once again contacted and the questionnaire was completed by telephone, in accordance with the method described by Peterson et al.^14^ All interviews were conducted by the same investigator, who wrote down and kept confidential all responses obtained, without expressing any judgment or comment. 

The data collected from medical records and interviews included patient age, parity, number of living children, contraceptive methods previously used and duration of use; and information on pre and post-sterilization menstrual cycles (menstruation duration, cycle interval, presence of intermenstrual bleeding, dysmenorrhea and amount of bleeding), premenstrual symptoms (edema, headache, nervousness and dizziness) and pelvic pain (noncyclic pain). Sexual performance (libido, dyspareunia and number of sexual intercourse occurrences per week) were investigated in accordance with the method proposed by Costello et al. This included the following questions: 1) Has tubal sterilization affected your sexual interest; that is, since your tubal sterilization operation, would you say you had less sexual interest, more, or about the same sexual interest compared with before the operation? and 2) Has tubal sterilization affected your pleasurable physical feelings during sexual relations; that is, since your tubal sterilization operation, would you say you had less, more, or about the same pleasurable feelings during sexual relations compared with before the operation?^15^ We also investigated the reason for choosing the surgical method, occurrences of post-sterilization regret, satisfaction level and whether the patients would recommend tubal ligation to others. In addition, other variables such as the presence of arterial hypertension, diabetes, cardiopathy and antecedents of deep vein thrombosis were evaluated.

To evaluate menstrual cycle intervals, menstrual cycles were considered to be regular when the intervals ranged from 24 to 35 days, or irregular when any interval outside this range or intermenstrual bleeding was observed. Menstrual bleeding for more than 7 days, and menstrual flow requiring the use of more than 6 sanitary pads/days, or 20 pads per menstrual period were considered to be abnormal.^9^

Self-reported dysmenorrhea was rated as (1) mild, when the discomfort was bearable and did not require use of medication; (2) moderate, when the pain required use of conventional medication; or (3) severe, when pain relief was not achieved through using conventional medication and administration of intravenous drugs in a hospital environment was necessary, or the pain and discomfort were severe enough to interfere with daily activities.^26^

The influence of length of time since surgery on the study parameters was analyzed by dividing the study population into three groups: Group I, up to two years after surgery; Group II, 2-5 years after surgery; and Group III, more than 5 years after surgery. In order to evaluate the effect of age at the time of sterilization on the study parameters, the age cutoff level was 35 years.

Tubal ligation was performed by means of bipolar electrocoagulation followed by tube sectioning or Yoon Silastic ring (Millennium Surgical Corporation, Montgomery, United States) placement by the same surgical team (Figure 1). For bipolar electrocoagulation and sectioning, the tube was grasped at a poorly vascularized area of the isthmus portion using 5 mm endoscopic bipolar forceps (Take-Apart; Karl Storz, Tuttlingen, Germany), so as not to cause great damage to the mesosalpinx. Intermittent cauterization of the tube was performed. The tissue destruction area was confined to 3-5 mm from the endoscopic forceps. Subsequently, the cauterized area was sectioned with a 5 mm hook scissor (Karl Storz). Careful hemostasis assessment was conducted after every procedure. The Silastic ring was applied using a surgical laparoscope (Olympus, Tokyo, Japan) containing two grasping forceps that drew a loop of tube into the outer cylinder of the ring applicator. This ring, which was preloaded stretched over the end of the cylinder, was then forced down over the loop, thereby releasing the portion of the tube encircled by the ring. 

The pre and post-sterilization variables were subjected to descriptive and numerical analysis using the Statistical Package for the Social Sciences (SPSS) software. Medians and the 25^th^ to 75^th^ percentile values for quantitative variables, and absolute frequencies and percentages for qualitative variables, were estimated. Wilcoxon's test for dependent samples was used to compare the pre and post-tubal ligation clinical characteristics, as well as the effects of Yoon Silastic ring placement versus electrocoagulation. To evaluate the effect of the operative technique on psychological characteristics, McNemar's test for dependent samples was used. Post-sterilization repercussions among the groups were analyzed by means of Fisher's exact test or the chi-square test. The significance level was set at 5% (P < 0.05). 

## RESULTS

Table 1 shows the clinical characteristics of the 130 patients included in this study. The median age was 33 years. Most women (82.3%) were married or in a stable relationship; 50% had up to three children; 63.1% had just had elementary education; and nearly 63% had a low family income. The most frequent comor bidities were smoking (22.3%) and hypertension (19.2%). 

**Table 1:** Simple and relative frequencies of the clinical and epidemiological characteristics of the patients who underwent videolaparoscopic tubal ligation (n = 130)

Variables	Values
Age at tubal ligation	33 (29-37)^*^
Parity (number of living children)	3.0 (2.0-4.0)^*^
Marital status	
Married	47.7% (n = 62)
Stable union	34.6% (n = 45)
Separated, widowed or single	17.7% (n = 23)
Schooling	
Elementary school	63.1% (n = 82)
High school	26.2% (n = 34)
University	10.0% (n = 13)
Illiterate	0.8% (n = 1)
Family income	
≤ 1 minimum wage	32.3% (n = 42)
2-3 minimum wages	30.8% (n = 40)
4-5 minimum wages	27.0% (n = 35)
> 5 minimum wages	10.0% (n = 13)
Smoking	22.3% (n = 29)
Hypertension	19.2% (n = 25)
Diabetes	2.3% (n = 3)
Cardiopathy	6.2% (n = 8)
Deep venous thrombosis	4.6% (n = 6)
Systemic lupus	3.1% (n = 4)

* Value expressed as medians (25th to 75th percentiles).

Occurrences of premenstrual symptoms such as headache, edema, mastalgia, dizziness and irritability before sterilization, which were reported by 55.4% of the patients, increased significantly to 74.6% after the procedure (P < 0.001). The rate of dysmenorrhea reported as moderate or incapacitating, which was 18.5% before tubal ligation, showed a significant increase to 13.8% afterwards (P = 0.019) Reported noncyclic pelvic pain also significantly increased from 12.3% pre-sterilization to 26.2% post-sterilization (P = 0.001). No difference in intermenstrual bleeding was observed after tubal ligation (Table 2). A significant difference in the 75^th^ percentile was observed in relation to use of sanitary pads, ranging from 5 to 8 pads on the day of heaviest flow (P < 0.001), and from 20 to 29 pads over the entire menstrual period (P = 0.001) (Table 3).

**Table 2:** Comparison between the occurrence of menstruation-related symptoms before and after videolaparoscopic tubal ligation (n = 130)

Variables	Pre-tubal ligation	Post-tubal ligation	P-value^*^
Premenstrual symptoms	55.4% (n = 72)	74.6% (n = 97)	< 0.001
Dysmenorrhea	18.5% (n = 24)	32.3% (n = 42)	0.019
Pelvic pain	12.3% (n = 16)	26.2% (n = 34)	0.001
Intermenstrual bleeding	13.8% (n = 18)	13.1% (n = 17)	1.000

Values expressed as percentages

* Significant difference if P < 0.05 (McNemar's test).

**Table 3:** Menstrual cycle, premenstrual symptoms and psychosexual characteristics among patients who underwent videolaparoscopic tubal ligation by means of Yoon Silastic' ring insertion (n = 50) or bipolar electrocoagulation and sectioning (n = 80)

Variables	Pre-tubal ligation Tubal ring	Post-tubal ligation Tubal ring	P-value^*^	Pre-tubal ligation Electrocoagulation	Post-tubal ligation Electrocoagulation	P-value^*^
Menstrual cycle (days)						
Minimum interval	28 (15-28)	25 (15-28)	0.978^(1)^	28 (20-28)	28 (20-28)	0.855^(1)^
Maximum interval	30 (30-30)	30 (30-35)	0.862^(1)^	30 (30-30)	30 (30-30)	0.514^(1)^
Length of bleeding (days)	5 (4-5)	5 (4-7)	0.235^(1)^	4 (3-5)	5 (3-6)	0.266^(1)^
Number of sanitary pads on day of heaviest flow	4 (3-5)	5 (3-8)	0.004^(1)^	4 (3-5)	4 (3-7)	0.002^(1)^
Number of sanitary pads used during menstruation	15 (10-20)	20 (10-29)	0.040^(1)^	11 (10-17)	15 (10-28)	0.015^(1)^
Intermenstrual bleeding	14.0% (n = 7)	12.0% (n = 6)	1.000^(2)^	12.5% (n = 10)	13.8% (n = 11)	1.000^(2)^
Spotting	6.0% (n = 3)	6.0% (n = 3)	1.000^(2)^	2.5% (n = 2)	5.0% (n = 4)	0.625^(2)^
Premenstrual symptoms	52.0% (n = 26)	72.0% (n = 36)	0.006^(2)^	55.0% (n = 44)	73.8% (n = 59)	0.006^(2)^
Noncyclic pelvic pain	14.0% (n = 7)	28.0% (n = 14)	0.092^(2)^	11.3% (n = 9)	23.8% (n = 19)	0.006^(2)^
Intercourse occurrences per week	3 (2-4)	3 (1-4)	0.045^(1)^	3 (2-4)	2 (1-3)	0.012^(1)^
Libido	76.0% (n = 38)	66.0% (n = 33)	0.302^(2) ^	78.8% (n = 63)	58.8% (n = 47)	0.001^(2)^
Dyspareunia	34.0% (n = 17)	22.0% (n = 11)	0.227^(2)^	17.5% (n = 14)	26.3% (n = 21)	0.092^(2)^

Values expressed as medians (25th to 75th percentile) and percentages

* Difference significant if P < 0.05 [(1)Wilcoxon's test for dependent samples; (2)McNemar's test]

Sexual life after sterilization was reported to be unchanged by 53% of the patients, and the reported rates of improvement and worsening were similar (23% and 24%, respectively). Nevertheless, the assessment of psychosexual symptoms showed that 16.2% of the women reported libido reduction after sterilization (P = 0.001), thus leading to a decrease in the average number of sexual intercourse occurrences per week: median (25^th^-50^th^ percentile) before = 3 (2-4); after = 2 (1-4); P = 0.001. The percentage of dyspareunia remained unchanged after tubal ligation (**Table 3**). Oral hormonal contraceptives (OHC) were the method last used before sterilization by 43% of the women, followed by condoms (22.3%), a intrauterine device (9.2%) and injectable hormonal contraceptives (8.5%). The study sample size did not allow statistical analysis of the influence of the contraceptive methods used before tubal ligation, on the study variables.

The operative technique used consisted of placement of a Yoon Silastic ring in 38.5% of the women and bipolar electrocoagulation plus sectioning of the uterine tube in 61.5%. The women who received a Yoon Silastic ring significantly more frequently reported menstrual flow increases, exacerbated premenstrual symptoms and lower numbers of sexual intercourse occurrences per week; whereas use of bipolar electrocoagulation and sectioning had an adverse influence on menstrual flow, premenstrual symptoms and number of sexual intercourse occurrences per week, and was also associated with increased noncyclic pelvic pain and libido reduction (Table 3). 

Taking into consideration the length of time that had elapsed since surgery, no significant differences were observed in the study parameters. On the other hand, age at the time of tubal ligation seemed to influence the post-sterilization clinical and psycho-sexual characteristics (Table 4). Using 35 years as a cutoff point, it was observed that among the women who underwent tubal ligation at this age or younger, the incidence of premenstrual symptoms, moderate and severe dysmenorrhea, noncyclic pelvic pain, heavier menstrual flow, libido reduction and decreased number of sexual intercourse occurrences/week was significantly higher after sterilization.

**Table 4:** Menstrual cycle, premenstrual symptoms and psychosexual characteristics of the women who underwent videolaparoscopic tubal ligation (n = 130), in relation to age at the time of surgery

Variables	Pre-tubal ligation	Post-tubal ligation	P-value^*^
Number of sanitary pads on day of heaviest flow			
≤ 35 years	4 (3-5)	5 (3-8)	< 0.001^(1)^
> 35 years	4 (3-5)	4 (3-7)	0.088^(1)^
Number of sanitary pads used during menstruation			
≤ 35 years	12 (10-20)	20 (10-35)	0.001^(1)^
> 35 years	15 (10-20)	15 (10-20)	0.433^(1)^
Premenstrual symptoms			
≤ 35 years	52.4%	75.6%	< 0.001^(2)^
> 35 years	60.9%	71.7%	0.227^(2)^
Dysmenorrhea			
≤ 35 years	19.0%	42.2%	0.011^(3)^
> 35 years	17.3%	15.2%	0.159^(3)^
Noncyclic pelvic pain			
≤ 35 years	11.9%	29.8%	0.001^(2)^
> 35 years	13.0%	19.6%	0.453^(2)^
Libido			
≤ 35 years	83.3%	59.5%	< 0.001^(2)^
> 35 years	69.6%	65.2%	0.754^(2)^
Intercourse occurrences per week			
≤ 35 years	3 (2-4)	2 (1-3)	0.001^(1)^
> 35 years	3 (2-4)	3 (2-4)	0.525^(1)^

Values expressed as medians (25th to 75th percentile) and percentages

* Difference significant if P < 0.05

(1) Wilcoxon Signed Rank test

(2) McNemar's test

(3) McNemar-Bowker test].

When questioned about the reasons for choosing surgical sterilization, 52.3% of the respondents mentioned the number of children that they already had and 30.8% a medical recommendation. Other causes were a traumatic experience from a previous birth, intolerance of other contraceptive methods, economic reasons, reliability of the surgical method and the women's ages. Only 7.7% of the participants reported regret; 10% would not choose this contraception method again; and 92.3% would recommend tubal ligation as a good contraception method to others. The hysterectomy rate observed after tubal ligation was only 5.4% and the causes were unrelated to this procedure.

## DISCUSSION

Over the past decades, advances in technology and anesthesiol ogy and improved surgical techniques have made female steril ization easier, faster and more effective. As a result, it is one of the most popular contraceptive choices for family planning. Between 1950 and 1980, the number of couples choosing tubal ligation as a birth control method increased from 3 million to 13 mil lion worldwide.31 In 1990, the proportion of married women of reproductive age using tubal ligation as a contraceptive method was 22% in developing countries, and 11% in developed coun tries, representing 44% and 18% of all contraceptive users in the respective countries.17 Today, tubal ligation is considered to be the most frequently performed and effective contraceptive proce dure, with a 0.4% pregnancy rate after one year and a cumulative failure rate of 1.85% after 10 years.^40^^,^ 41 However, these rates may vary according to the age of the patient at the time of tubal liga tion and the technique used in the procedure.^18^

The Brazilian National Survey on Demography and Health (PNDS) conducted in 1996 showed that 40.1% of the women aged 15-49 years who were in a stable relationship had undergone tubal ligation. Their mean age at the time of tubal ligation was 28.9 years, and 57% of the women had undergone sterilization before the age of 30 years.^42^^-^ 44 According to the latest PNDS, conducted in 2006, a reduction to 29% of those committed to a stable relationship was observed, with a mean age at the time of tubal ligation of 27.1 years, and 63.4% of the women had undergone sterilization before the age of 30 years.^2^ In the United States, according to the U.S. National Survey of Family Growth carried out in 2002, 23.2% of the women aged 15-44 years using a birth control method chose tubal ligation, and according to the numbers of the 2006-2008 survey, 21.1% of married women reported having undergone tubal sterilization.^18^^,^ 45

Questions regarding the existence of a post-tubal ligation syndrome arose as early as 1951, before regulations for this practice were established in Brazil. Despite the numerous studies conducted, the various types of study design used, the different factors investigated (clinical, laboratory or imaging) and, above all, the considerable number of women observed, no consensus has been reached. 

This study showed that tubal ligation had an adverse influence on menstrual flow, premenstrual symptoms and weekly frequency of sexual intercourse, regardless of the operative technique used. Electrocoagulation, which causes greater tissue damage than Silastic ring placement, also had an adverse effect on reported noncyclic pelvic pain and libido. This finding is in agreement with Shain et al., who observed clear changes in menstrual patterns among women undergoing tubal ligation by means of electrocoagulation, compared with Silastic ring insertion.^33^ Nonetheless, other investigators did not find any significant differences according to the method of sterilization.^14^^,^ 46

Menstrual flow, as estimated by the number of sanitary pads used on the day of heaviest flow and throughout menstruation, varied from 5 to 8 pads on the day of heaviest flow, and from 20 to 29 throughout menstruation at the 75^th^ percentile in the whole sample, i.e. regardless of the operative technique used. In the United States, the CREST study (Collaborative Review of Sterilization) found that there was a significant increase in menstrual flow among 5,070 women within five years after tubal ligation.^25^ MacKenzie et al. also observed that the frequency of heavy periods increased from 9% before to 35% after sterilization with Filshie clips.^47^ In turn, Peterson et al. reported that there were no changes in menstrual flow in women who had undergone tubal ligation, in comparison with a control group consisting of women whose husbands had had vasectomy. In fact, they found that the women who experienced heavy vaginal bleeding before sterilization presented significantly reduced bleeding.^14^ Other investigators also found no association between tubal ligation and changes in menstrual pattern including intermenstrual bleeding, cycle interval duration and length or amount of bleeding.^4^^,^ 36

The rate of reported adverse premenstrual symptoms (headache, edema, mastalgia, dizziness and irritability) increased from 55.4% to 74.6% after sterilization, regardless of the operative technique used. Reported dysmenorrhea also increased significantly (from 18.5% to 32.3%), as well as noncyclic pelvic pain (from 12.3% to 26.2%), in agreement with the study by MacKenzie et al.^47^ Morrissey et al. suggested that retrograde menstruation dilating the proximal tubal stumps might be the potential cause of dysmenorrhea.^41^ However, our findings disagree with those reported by several authors who found no significant changes in the abovementioned variables.^9^^,^ 25 ^,^ 48

Sexual life after sterilization was reported to be unchanged among 53% of our patients, and the reported rates of improvement and worsening were similar (23% and 24%, respectively), thus suggesting that tubal ligation had no influence on sexual performance. In the literature, there are reports of increases in sexual satisfaction of 6-55% and decreases of 0-7%.^6^^,^ 8 ^,^ 11 ^,^ 15 Nonetheless, our results demonstrate that there was a significant drop in the number of intercourse occurrences per week, as well as a reduction in libido, and these changes were more evident among the women undergoing tubal ligation by means of bipolar electrocoagulation. Other studies have demonstrated occurrences of libido reduction in 2-5% of the cases and increases in 21-25%.^7^^,^ 11 ^,^ 15 ^,^ 48

In the present study, the analysis on changes to sexual performance and the assessment of the mean number of intercourse occurrences per week, both before and after sterilization, suggest that rather than being related to tubal ligation, the reduction in sexual intercourse frequency is influenced by other factors such as the status of marital relations, which was reported to be regular, conflicting or bad by 37.7% of the respondents. This finding is corroborated by Costello et al., who found that 80% of the 4576 women participating in the CREST study reported that there were no consistent changes in sexual interest and pleasure after sterilization. Among the women who reported consistent changes in their sexual life, positive effects, i.e. increased sexual interest and pleasure, were reported to be 10 and 15-fold greater, respectively.^15^

Age at the time of tubal ligation influenced the study parameters, especially among women who underwent sterilization at the age of 35 years or less. Shobeiri et al. found that the highest frequency of menstrual abnormalities, characterized by polymenorrheic cycles, was 54.3% for the group aged between 30 and 39 years, in comparison with a control group consisting of women whose husbands had undergone vasectomy.^17^ Shy et al. suggested that the effect of sterilization on menstrual changes depends on age at the time of sterilization, since women who undergo sterilization between 20 and 29 years of age have more menstrual irregularities than women who undergo the procedure after the age of 30 years.^34^ On the other hand, Rulin et al. reported that there were significant changes only among patients older than 35 years.^10^

In contrast with other studies, the length of time since surgery did not have any influence on the clinic parameters assessed here. According to Stock et al., changes in menstrual pattern and pelvic pain most frequently occurred within eight months after tubal ligation.^5^ DeStefano et al. demonstrated that menstrual changes increased with time and became more evident after two years.^9^ In contrast, some studies reported that there was a progressive decrease in menstrual changes as time went by.^17^^,^ 47 ^,^ 48

It has been estimated that the regret rate after tubal sterilization among women in the general population ranges from 10% to 20%.^39^^,^ 43 ^,^ 44 ^,^ 49 ^,^ 50 Dias et al. observed a regret rate of 6.7%, predominantly among women who underwent tubal ligation at a young age.^12^ Regret could be a major cause of intolerance of symptoms after tubal ligation, thus increasing the number of reports in this specific group. According to the CREST study, which followed sterilized women for up to 14 years after their tubal ligation, the cumulative probability of expressing regret following tubal sterilization was 12.7% among all the women assessed: 5.9% among those older than 30 years, and 20.3% among women aged 30 years or less at the time of tubal ligation.^18^ In our study, a 7.7% regret rate was observed and 10% of the women would not choose this contraceptive method again. The great majority were satisfied with the procedure and 92.3% of the respondents would recommend tubal ligation as a good method to others.

Regret was reported to be due to changes in the menstrual pattern, such as great increases in bleeding, noncyclic abdominal pain and dysmenorrhea, in 20% of the cases. It is noteworthy that 40% of the cases of regret were associated with a medical indication for which strict criteria should be followed in order to avoid iatrogenic effects such as those described by Costello et al., who demonstrated that post-sterilization regret was the only indicator for decreased sexual interest and sexual pleasure; or those reported by Fernandes et al., who found a success rate of only 6% among patients desiring tubal ligation reversal.^15^^,^ 51 It can be noted that young women are more susceptible to changes in their lifestyle and new family unions, which inevitably lead them to the desire for new pregnancies and an increased risk of regret after tubal ligation.

The CREST study found a five-year cumulative probability of hysterectomy that was four to fivefold higher in sterilized women, compared with women whose partner had had a vasectomy.^18^ However, another recent study showed that tubal sterilization is not a risk factor for undergoing hysterectomy.^52^ The low hysterectomy rate found here (5.4%) does not seem to be associated with sterilization, since all but one patient had well defined diagnoses, not related to sterilization, that justified the procedure. This finding goes against the existence of the so called post-tubal ligation syndrome as described by Williams and others.^3^^,^ 22 ^,^ 27

The outcomes were surveyed between 12 and 119 months after the sterilization procedure, but no individual analysis on this parameter was made, or on the mean time of investigation, which led to omission of these data. However, to evaluate the time that had elapsed since sterilization in relation to the study parameters, the patients were divided into three groups: Group I, up to 2 years after surgery; Group II, 2-5 years after surgery; and Group III, more than 5 years after surgery. No significant differences were observed between these groups. 

It is also important to emphasize that one of the main weaknesses of this study was the absence of a control group and the subjectivity of the patients' complaints. To minimize this effect, we sought to make evaluations in the most straightforward way as possible, like counting the number of sanitary pads used on the day of heaviest flow and during the entire menstrual period to quantify bleeding; and evaluating the impact of the symptoms on the daily activities of each woman surveyed. Prospective analysis of objective parameters in relation to a matched control group might help to clarify some of the issues presenting controversy in the literature.

Choosing tubal ligation shows a strong desire for effective birth control and family planning. Indeed, several factors may lead a woman to choose a permanent contraceptive method. One of them is the belief that an operative method is more effective than any other reversible procedure, despite the numerous studies demonstrating that other methods, when correctly used, have similar effectiveness. The relationship context within which sterilization decisions are made has changed considerably. Today's women, especially those who are single or in non-stable relationships, are increasingly likely to make the decision to seek sterilization on their own.^53^^,^ 54 Thus, resolving the debate on menstrual changes relating to post-tubal ligation is important for women's health and welfare, particularly in a country where millions undergo this contraceptive procedure every year. 

Less invasive techniques such as hysteroscopic sterilization are now increasingly gaining ground, but so far there have not been any studies on their impact on the physical and psychosexual wellbeing of women.^55^^,^ 56 According to Peterson et al., menstrual abnormalities and tubal ligation are common, and are therefore likely to occur coincidentally.^14^ As stated by Gentile et al., whatever the explanation, the point is that rather than avoiding tubal ligation because it might be associated with menstrual abnormalities, women should be informed about the possibility of menstrual changes after the procedure, the regret rate, psychosexual dissatisfaction and other risks, as well as the benefits of this kind of surgery.^25^ Whenever menstrual and sexual abnormalities occur after tubal ligation, physicians should remember to discuss with their patients the possible influence of this procedure on their symptoms. 

The potential relationship of clinical outcomes after tubal ligation with the regret experienced by some women suggests that there is a need for rigorous and well-defined criteria in applying a definitive contraception method. Analysis of specific populations with and without regrets may elucidate the impact of psychoemotional factors on the clinical parameters reported.

## CONCLUSION

Regardless of the technique used, videolaparoscopic tubal liga tion was associated with increased amounts of bleeding and pre menstrual distress, especially among women aged â‰¤ 35 years, besides having an adverse influence on sexual activity. Despite this, the overall satisfaction with the procedure was 92.3%. 
